# Pattern of Musculoskeletal Pain Among Patients With Type 2 Diabetes: A Cross-Sectional Study

**DOI:** 10.7759/cureus.113712

**Published:** 2026-07-31

**Authors:** Mohammed Mohsin Raza, Sana Sajjad, Wardah Ikram, Abdul Manan, Shehriyar Khan, Mutea Ullah

**Affiliations:** 1 Department of Medicine, Unit 3, Shaikh Zayed Medical College and Hospital, Rahim Yar Khan, PAK; 2 Department of Internal Medicine, Allama Iqbal Medical College, Lahore, PAK; 3 Department of General Internal Medicine, Barts Health NHS Trust, London, GBR; 4 Department of Internal Medicine, Saidu Teaching Hospital, Swat, PAK; 5 Department of Internal Medicine, University Hospitals Birmingham NHS Foundation Trust, Birmingham, GBR; 6 Department of Internal Medicine, Hayatabad Medical Complex, Peshawar, PAK

**Keywords:** diabetic complications, knee pain, low back pain, musculoskeletal pain, neuropathy, obesity, shoulder pain, type 2 diabetes mellitus

## Abstract

Background and objective

Musculoskeletal pain (MSKP) is a common but frequently underrecognized complication among patients with type 2 diabetes mellitus (T2DM). Chronic hyperglycemia, obesity, peripheral neuropathy, reduced physical activity, microvascular complications, and altered biomechanics have all been implicated in the development of musculoskeletal disorders affecting the spine, shoulder, knee, hand, and foot. The objective of this study is to determine the frequency, anatomical pattern, and severity of MSKP and to identify the clinical factors associated with MSKP among patients with T2DM.

Methods

A descriptive cross-sectional study was conducted among 230 consecutive adult patients with T2DM attending the outpatient diabetes clinic of Sheikh Zayed Hospital, Rahim Yar Khan, Pakistan. Demographic characteristics, duration of diabetes, body mass index (BMI), glycemic control (HbA1c), diabetic complications, physical activity, and MSKP characteristics were recorded using a structured interviewer-administered questionnaire and review of medical records. Pain was assessed across eight anatomical regions using a structured body-region questionnaire, and pain severity was graded using the 11-point Numerical Rating Scale (NRS). Data were analyzed using descriptive statistics and the chi-square test, with a p-value <0.05 considered statistically significant.

Results

The prevalence of MSKP was 73.0% (168/230). The mean age of participants was 55.8 ± 10.6 years, and 129 (56.1%) were female. The lower back was the most frequently affected site (101, 43.9%), followed by the shoulder (83, 36.1%), knee (76, 33.0%), neck (58, 25.2%), hand/wrist (48, 20.9%), ankle/foot (42, 18.3%), hip (34, 14.8%), and elbow (20, 8.7%). Multiple-site pain was reported by 97 (57.7%) patients with MSKP, while moderate pain was the most common severity category (96, 41.7%). MSKP was significantly associated with female sex, longer duration of diabetes (>10 years), obesity, poor glycemic control (HbA1c ≥8%), peripheral neuropathy, and low physical activity (all p < 0.05).

Conclusion

MSKP is highly prevalent among patients with T2DM, with the lower back, shoulder, and knee being the most commonly affected anatomical sites. Female sex, longer diabetes duration, obesity, poor glycemic control, peripheral neuropathy, and low physical activity were significantly associated with MSKP. Routine musculoskeletal assessment should be integrated into comprehensive diabetes care to facilitate early identification, multidisciplinary management, and improved physical function and quality of life.

## Introduction

The prevalence, morbidity, and burden of type 2 diabetes mellitus (T2DM) have grown to significant levels globally. The International Diabetes Federation has highlighted the growing global burden of diabetes, and current diabetes care guidelines have focused on ensuring diabetes is managed beyond the management of glucose alone [1,2]. Diabetic complications, such as retinopathy, nephropathy, neuropathy, and cardiovascular complications, are the focus of much clinical attention; however, musculoskeletal complications are also common and clinically significant [3,4].

Diabetes-related musculoskeletal disorders include adhesive capsulitis, limited joint mobility, carpal tunnel syndrome, trigger finger, Dupuytren’s contracture, osteoarthritis, diffuse idiopathic skeletal hyperostosis, Charcot arthropathy, and generalized musculoskeletal pain (MSKP) [3-6]. These conditions may occur because of advanced glycation end-products, connective tissue stiffening, chronic inflammation, microvascular disease, neuropathy, and altered biomechanics associated with obesity and physical inactivity [5,7].

Previous studies have reported that MSKP is more common among patients with type 2 diabetes than among non-diabetic populations and that pain may impair physical function, quality of life, and adherence to exercise-based diabetes management [6,8]. Shoulder, lumbar spine, knee, ankle, and hand pain have been reported as common sites in diabetic populations [8-12]. However, local data regarding the pattern of MSKP among patients with type 2 diabetes remain limited. This study was therefore conducted to determine the frequency, anatomical pattern, severity, and associated clinical factors of MSKP among 230 patients with T2DM.

## Materials and methods

Study design and setting

A descriptive cross-sectional study was conducted at the outpatient diabetes clinic of Sheikh Zayed Hospital, Rahim Yar Khan, Pakistan, between October 2025 and March 2026. The study aimed to determine the prevalence, anatomical distribution, severity, and clinical factors associated with MSKP among patients with T2DM.

Study population

The required sample size was calculated using OpenEpi (Version 3.01) based on the single population proportion formula. Assuming a prevalence of 24% for musculoskeletal disorders among patients with diabetes mellitus, as reported by Muluneh et al. [11], a 95% confidence level, a 6% margin of error, and a 10% allowance for non-response, the minimum required sample size was estimated to be 215 participants. A total of 230 consecutive adult patients with established T2DM attending the outpatient diabetes clinic during the study period were therefore enrolled. Consecutive recruitment was achieved by inviting every eligible patient attending the outpatient diabetes clinic during the study period until the required sample size was reached.

Patients were eligible if they were aged 30 years or older, had a confirmed diagnosis of T2DM for at least one year, and provided written informed consent. Patients with T1DM, recent musculoskeletal trauma, known inflammatory arthritis, malignancy, pregnancy, chronic kidney disease requiring dialysis, or a history of major orthopedic surgery were excluded to minimize potential confounding causes of MSKP.

Data collection

Data were collected using a structured interviewer-administered questionnaire together with a review of patients' medical records. The questionnaire was developed based on a review of the relevant literature and was reviewed by two consultant physicians for content relevance and clarity before use. A pilot test was conducted among 15 patients with T2DM who were not included in the final analysis to ensure comprehensibility and feasibility. Information collected included demographic characteristics (age and sex), clinical characteristics (body mass index (BMI), duration of diabetes, treatment modality, glycated hemoglobin (HbA1c), hypertension, peripheral neuropathy, retinopathy, and physical activity status), and MSKP characteristics. Data were collected by trained investigators using a standardized data collection procedure. The structured study questionnaire used for data collection is provided in Appendix 1. Questionnaires were checked for completeness at the time of data collection. Records with missing information on the primary outcome or key study variables were excluded from analysis; consequently, no imputation of missing data was performed.

MSKP was defined as pain, aching, stiffness, or discomfort involving muscles, joints, or periarticular structures experienced during the preceding three months. Pain was assessed at eight anatomical sites, including the neck, shoulder, elbow, hand/wrist, lower back, hip, knee, and ankle/foot. Patients reporting pain at more than one anatomical site were categorized as having multiple-site pain. Pain severity was assessed using the 11-point Numerical Rating Scale (NRS), where 0 indicated no pain and 10 indicated the worst imaginable pain. Pain intensity was categorized as mild (1-3), moderate (4-6), and severe (7-10). Peripheral neuropathy and diabetic retinopathy were identified from documented clinical diagnoses in the patients' medical records. HbA1c values were obtained from the most recent laboratory measurement performed within three months before study enrollment.

Statistical analysis

Data were entered and analyzed using IBM SPSS Statistics for Windows, Version 27 (Released 2019; IBM Corp., Armonk, NY, USA). Continuous variables were summarized as mean ± standard deviation (SD), while categorical variables were presented as frequencies and percentages. Associations between MSKP and categorical clinical variables were evaluated using the chi-square test. A two-sided p-value <0.05 was considered statistically significant.

## Results

A total of 230 patients with T2DM were enrolled, with a mean age of 55.8 ± 10.6 years. The largest proportion belonged to the 51-60-year age group (87, 37.8%), and females slightly outnumbered males (129, 56.1%). More than one-third of participants had diabetes for over 10 years (86, 37.4%), while oral hypoglycemic agents alone were the most common treatment modality (112, 48.7%). Overall, 118 (51.3%) patients had poor glycemic control (HbA1c ≥8%), 132 (57.4%) had hypertension, 74 (32.2%) had peripheral neuropathy, and 61 (26.5%) had diabetic retinopathy. Additionally, 78 (33.9%) participants were obese, and 157 (68.3%) reported low or irregular physical activity (Table 1).

**Table 1:** Baseline demographic and clinical characteristics of the study participants (N = 230)

Variable	n (%)
Age group (years)	
30-40	22 (9.6)
41-50	51 (22.2)
51-60	87 (37.8)
>60	70 (30.4)
Sex	
Male	101 (43.9)
Female	129 (56.1)
Duration of diabetes	
1-5 years	78 (33.9)
6-10 years	66 (28.7)
>10 years	86 (37.4)
Body mass index (BMI)	
Normal weight	42 (18.3)
Overweight	110 (47.8)
Obese	78 (33.9)
Treatment modality	
Oral hypoglycemic agents only	112 (48.7)
Insulin only	36 (15.7)
Oral hypoglycemic agents + insulin	82 (35.6)
HbA1c level	
<8%	112 (48.7)
≥8%	118 (51.3)
Hypertension	
Present	132 (57.4)
Absent	98 (42.6)
Peripheral neuropathy	
Present	74 (32.2)
Absent	156 (67.8)
Diabetic retinopathy	
Present	61 (26.5)
Absent	169 (73.5)
Physical activity	
Regular	73 (31.7)
Low/Irregular	157 (68.3)

MSKP was reported by 168 (73.0%) patients. Among those with MSKP, moderate pain was the most common severity category (96, 41.7%), and multiple-site pain was more frequently reported than single-site pain (97, 57.7% vs. 71, 42.3%) (Table 2).

**Table 2:** Characteristics of MSKP among patients with T2DM (N = 230) *Calculated among patients with MSKP (n = 168). T2DM: type 2 diabetes mellitus; MSKP: musculoskeletal pain

Variable	n (%)
Overall MSKP	
Present	168 (73.0)
Absent	62 (27.0)
Pain severity	
Mild	42 (18.3)
Moderate	96 (41.7)
Severe	30 (13.0)
Number of painful sites*	
Single-site	71 (42.3)
Multiple-site	97 (57.7)

The lower back was the most frequently affected anatomical site (101, 43.9%), followed by the shoulder (83, 36.1%) and knee (76, 33.0%), whereas elbow pain was the least commonly reported (20, 8.7%) (Table 3). The predominance of lower back pain is consistent with its high prevalence in both diabetic and general populations. However, as this study was descriptive in nature, no attempt was made to determine the specific anatomical or biomechanical source of low back pain, such as lumbar spine or sacroiliac joint pathology. Therefore, the observed predominance of lower back pain should be interpreted as a descriptive finding rather than evidence of a particular underlying pathology.

**Table 3:** Anatomical distribution of musculoskeletal pain (N = 230) Some patients had pain at more than one anatomical site; therefore, percentages exceed 100 when combined.

Pain site	n (%)
Lower back	101 (43.9)
Shoulder	83 (36.1)
Knee	76 (33.0)
Neck	58 (25.2)
Hand/Wrist	48 (20.9)
Ankle/Foot	42 (18.3)
Hip	34 (14.8)
Elbow	20 (8.7)

MSKP was significantly associated with female sex (χ² = 4.39, p = 0.036), longer duration of diabetes (χ² = 9.04, p = 0.011), higher BMI (χ² = 9.66, p = 0.008), poor HbA1c (χ² = 7.55, p = 0.006), peripheral neuropathy (χ² = 9.47, p = 0.002), and low physical activity (χ² = 8.89, p = 0.003) (Table 4). Among these, the highest prevalence of MSKP was observed in patients with peripheral neuropathy (64, 86.5%), obesity (66, 84.6%), HbA1c ≥8% (96, 81.4%), and diabetes duration >10 years (71, 82.6%).

**Table 4:** Unadjusted associations between musculoskeletal pain and participant characteristics *p-value <0.05 was significant.

Variable	Pain present n (%)	Pain absent n (%)	χ²	p-value*
Sex				
Male	67 (66.3)	34 (33.7)	4.39	0.036
Female	101 (78.3)	28 (21.7)		
Duration of diabetes				
1-5 years	48 (61.5)	30 (38.5)	9.04	0.011
6-10 years	49 (74.2)	17 (25.8)		
>10 years	71 (82.6)	15 (17.4)		
Body mass index				
Normal	24 (57.1)	18 (42.9)	9.66	0.008
Overweight	78 (70.9)	32 (29.1)		
Obese	66 (84.6)	12 (15.4)		
Treatment modality				
Oral medications only	77 (68.8)	35 (31.2)	2.84	0.241
Insulin only	29 (80.6)	7 (19.4)		
Oral medications + insulin	62 (75.6)	20 (24.4)		
HbA1c				
<8%	72 (64.3)	40 (35.7)	7.55	0.006
≥8%	96 (81.4)	22 (18.6)		
Hypertension				
Present	101 (76.5)	31 (23.5)	1.74	0.187
Absent	67 (68.4)	31 (31.6)		
Peripheral neuropathy				
Present	64 (86.5)	10 (13.5)	9.47	0.002
Absent	104 (66.7)	52 (33.3)		
Diabetic retinopathy				
Present	50 (82.0)	11 (18.0)	3.18	0.074
Absent	118 (69.8)	51 (30.2)		
Physical activity				
Regular	43 (58.9)	30 (41.1)	8.89	0.003
Low/Irregular	125 (79.6)	32 (20.4)		

## Discussion

This study found a high prevalence of MSKP among patients with T2DM, with nearly three-quarters of the study population reporting pain. This finding is consistent with previous studies showing that MSKP and disability are common among diabetic patients and may be more frequent than in the general population [11-13]. The high prevalence observed in this study may be explained by the combined effects of chronic hyperglycemia, obesity, peripheral neuropathy, reduced physical activity, chronic low-grade inflammation, and degenerative musculoskeletal changes associated with diabetes.

Lower back pain was the most frequently reported site in the present study. This finding agrees with studies demonstrating a high burden of low back pain among people with diabetes, particularly those with type 2 diabetes [13]. The mechanisms underlying low back pain in this population are likely multifactorial and may include obesity, paraspinal muscle dysfunction, neuropathic pain, disc degeneration, systemic inflammation, and altered biomechanics associated with diabetes [14,15]. In addition, increased mechanical loading across the pelvis during upright posture may place substantial stress on the sacroiliac joints, which have also been recognized as a potential source of chronic low back pain. Bertrand et al. reported that sacroiliac asymmetry was common among patients with chronic low back pain and suggested that assessment of sacroiliac joint dysfunction may have clinical value in selected patients, although further validation is required [16]. Our study did not include clinical examination, biomechanical assessment, or imaging to differentiate sacroiliac joint dysfunction from lumbar spine pathology, so the observed predominance of lower back pain should be interpreted as a descriptive finding rather than evidence of a specific anatomical source.

Shoulder pain was the second most common site in this study. Diabetes is a well-established risk factor for adhesive capsulitis and reduced shoulder range of motion [17,18]. Long-standing hyperglycemia promotes the accumulation of advanced glycation end products, resulting in collagen cross-linking, connective tissue stiffness, and reduced capsular elasticity, which may contribute to painful restriction of shoulder movement. Shoulder involvement is clinically important because it can adversely affect daily activities, sleep quality, self-care, and adherence to exercise-based diabetes management.

Knee pain was also common in the current study. The association between type 2 diabetes and osteoarthritis has been reported in systematic reviews and meta-analyses [19,20]. Obesity is a shared risk factor for both diabetes and knee osteoarthritis; however, metabolic inflammation and advanced glycation may also contribute independently to cartilage damage and pain. In this study, obese patients had a higher frequency of MSKP, supporting the role of excess body weight and mechanical loading.

Hand and wrist pain were reported in approximately one-fifth of patients. Diabetes-related hand conditions include limited joint mobility, carpal tunnel syndrome, trigger finger, and Dupuytren’s contracture [21-23]. Carpal tunnel syndrome has been associated with diabetes in previous meta-analyses, while trigger finger is also recognized as more common among diabetic patients [24,25]. These hand problems may reduce grip strength, impair fine motor function, and affect quality of life.

In the present study, poor glycemic control was a significant risk factor for MSKP. This finding further supports the concept that chronic hyperglycemia contributes to musculoskeletal complications through connective tissue glycation, collagen cross-linking, tendon stiffness, microvascular dysfunction, chronic low-grade inflammation, and neuropathic changes. Obesity, which frequently coexists with poor glycemic control, may further increase mechanical loading on weight-bearing joints of the lower limbs and feet, thereby contributing to pain and functional impairment. Furthermore, a longer duration of diabetes (>10 years) was associated with a higher frequency of MSKP, suggesting that the cumulative effects of prolonged metabolic dysregulation and tissue damage increase the risk of musculoskeletal complications over time.

Peripheral neuropathy was another significant factor associated with MSKP. Neuropathy may contribute to burning pain, altered sensation, gait abnormalities, abnormal joint loading, and foot and ankle disorders, thereby increasing susceptibility to musculoskeletal symptoms. In addition, pain often reduces physical activity, creating a vicious cycle in which inactivity contributes to poorer glycemic control, progressive weight gain, reduced muscle strength, and further musculoskeletal dysfunction [26]. Emerging evidence also suggests that dietary patterns associated with obesity and chronic hyperglycemia may influence gut microbiota composition and systemic inflammation. However, because dietary habits and gut microbiota were not evaluated in the present study, their potential contribution remains speculative and warrants further investigation.

The findings of this study emphasize the importance of incorporating routine musculoskeletal assessment into the comprehensive care of patients with type 2 diabetes. Clinical evaluation should include the location, severity, duration, and functional impact of pain, together with assessment for neuropathic symptoms and limitations in physical activity. Early multidisciplinary management - including patient education, optimization of glycemic control, weight management, individualized exercise programs, physiotherapy, appropriate analgesia, and referral to orthopedic or rheumatology specialists when indicated - may help reduce pain, improve function, and enhance quality of life.

Limitations

This study has several limitations. First, its cross-sectional design precludes the establishment of causal relationships, and the identified variables should therefore be interpreted as associated factors rather than risk factors. Second, participants were recruited consecutively from a single outpatient diabetes clinic, which may have introduced selection bias and limits the generalizability of the findings to other healthcare settings and populations. Third, the absence of a non-diabetic comparison group prevented direct comparison of the prevalence and pattern of MSKP between diabetic and non-diabetic individuals. Fourth, pain characteristics and physical activity were based on patient self-report and may therefore be subject to recall and reporting bias. In addition, although a structured questionnaire was used, its psychometric properties were not formally evaluated. Finally, musculoskeletal conditions and the anatomical source of pain were not routinely confirmed by radiological, electrophysiological, or specialized clinical assessments; therefore, specific pathologies, including lumbar spine and sacroiliac joint disorders, could not be distinguished.

## Conclusions

MSKP represents an important yet frequently overlooked complication of T2DM that has the potential to adversely affect physical function, mobility, treatment adherence, and overall quality of life. Routine assessment of musculoskeletal symptoms should therefore be incorporated into comprehensive diabetes care to facilitate early recognition and timely multidisciplinary management, including patient education, optimization of glycemic control, lifestyle modification, weight management, individualized exercise programs, physiotherapy, and appropriate specialist referral when indicated.

Greater awareness among healthcare professionals regarding diabetes-related musculoskeletal disorders may help reduce disability and improve long-term patient outcomes. Future multicenter prospective studies involving larger and more diverse populations are warranted to further investigate the mechanisms underlying MSKP, including the contributions of metabolic abnormalities, obesity-related biomechanical loading, neuropathy, systemic inflammation, and potential site-specific pathologies such as sacroiliac joint dysfunction. Such studies should also incorporate site-specific pain assessment, standardized clinical evaluation, and longitudinal follow-up to better define temporal relationships and inform effective preventive and therapeutic strategies for musculoskeletal complications in patients with T2DM.

## Appendices

**Table 5:** Structured questionnaire used for assessment of musculoskeletal pain pattern among patients with type 2 diabetes mellitus Credits: Mohammed Mohsin Raza, Sana Sajjad, Wardah Ikram, Abdul Manan, Shehriyar Khan, and Mutea Ullah

Section	Variable/Question	Response Options / Measurement
Patient Information	Study ID	__________
	Date	___ / ___ / _____
	Hospital	Sheikh Zayed Hospital, Rahim Yar Khan
	Interviewer	__________________
	Age (years)	____ years
	Age Group	30-40; 41-50; 51-60; >60 years
	Gender	Male; Female
	Height	____ cm
	Weight	____ kg
	BMI (kg/m²)	____ kg/m²
	BMI Category	Normal (<25); Overweight (25-29.9); Obese (≥30)
Diabetes Profile	Duration of Type 2 Diabetes	1-5 years; 6-10 years; >10 years
	Current Diabetes Treatment	Oral medicines only; Insulin only; Oral medicines + Insulin
	Latest HbA1c	____ %
	HbA1c Category	<8%; ≥8%
Medical History	Hypertension	Yes; No
	Peripheral Neuropathy (Diagnosed)	Yes; No
	Diabetic Retinopathy	Yes; No
	Other Chronic Illness	None; Osteoarthritis; Rheumatoid arthritis; Others (Specify)
Physical Activity	Physical Exercise (usual week)	Regular (≥150 min/week); Irregular/Low (<150 min/week)
Musculoskeletal Pain Screening	Pain, stiffness, or aching during the last 3 months	Yes; No (If No, proceed to Pain Severity section)
Pattern of Musculoskeletal Pain	Pain Location	Neck; Shoulder; Elbow; Hand/Wrist; Lower Back; Hip; Knee; Ankle/Foot (Yes/No for each site)
	Number of Painful Sites	One; Two; Three or more
	Duration of Pain	<1 month; 1–3 months; >3 months
	Nature of Pain	Aching; Sharp; Burning; Stiffness; Other (Specify)
	Pain Occurs	At rest; During movement; Both
	Pain Disturbs Sleep	Yes; No
	Pain Interferes with Daily Activities	Yes; No
Pain Severity (Numerical Rating Scale)	Average Pain Score	0–10 Numerical Rating Scale (0 = No pain; 10 = Worst imaginable pain)
	Pain Category	No Pain (0); Mild (1–3); Moderate (4–6); Severe (7–10)
Functional Limitation	Difficulty due to Pain	Walking; Climbing stairs; Lifting objects; Dressing; Household work; Sleeping (Yes/No for each activity)
Pain Medication	Use of Medication for Musculoskeletal Pain	No; Occasionally; Regularly
Clinical Examination (Investigator)	Height	____ cm
	Weight	____ kg
	BMI	____ kg/m²
	HbA1c	____ %
	Neuropathy	Present; Absent
	Retinopathy	Present; Absent
SPSS Coding	Gender	Male = 1; Female = 2
	Musculoskeletal Pain	No = 0; Yes = 1
	Neck Pain	No = 0; Yes = 1
	Shoulder Pain	No = 0; Yes = 1
	Elbow Pain	No = 0; Yes = 1
	Hand/Wrist Pain	No = 0; Yes = 1
	Low Back Pain	No = 0; Yes = 1
	Hip Pain	No = 0; Yes = 1
	Knee Pain	No = 0; Yes = 1
	Ankle/Foot Pain	No = 0; Yes = 1
	Pain Severity	None = 0; Mild = 1; Moderate = 2; Severe = 3
	Physical Activity	Regular = 1; Low = 2
	Peripheral Neuropathy	No = 0; Yes = 1
	Diabetic Retinopathy	No = 0; Yes = 1
	Hypertension	No = 0; Yes = 1
